# Mitochondrial Quality Control in Age-Related Pulmonary Fibrosis

**DOI:** 10.3390/ijms21020643

**Published:** 2020-01-18

**Authors:** Willy Roque, Karina Cuevas-Mora, Freddy Romero

**Affiliations:** 1 Department of Medicine, Rutgers New Jersey Medical School, 185 S Orange Ave, Newark, NJ 07103, USA; wr160@njms.rutgers.edu; 2 Center for Translational Medicine and Jane and Leonard Korman Lung Center, Thomas Jefferson University, Philadelphia, PA 19107, USA; karina.cuevas.mora@jefferson.edu

**Keywords:** mitochondria, quality control, mitophagy, proteostasis, biogenesis

## Abstract

Idiopathic pulmonary fibrosis (IPF) is age-related interstitial lung disease of unknown etiology. About 100,000 people in the U.S have IPF, with a 3-year median life expectancy post-diagnosis. The development of an effective treatment for pulmonary fibrosis will require an improved understanding of its molecular pathogenesis and the “normal” and “pathological’ hallmarks of the aging lung. An important characteristic of the aging organism is its lowered capacity to adapt quickly to, and counteract, disturbances. While it is likely that DNA damage, chronic endoplasmic reticulum (ER) stress, and accumulation of heat shock proteins are capable of initiating tissue repair, recent studies point to a pathogenic role for mitochondrial dysfunction in the development of pulmonary fibrosis. These studies suggest that damage to the mitochondria induces fibrotic remodeling through a variety of mechanisms including the activation of apoptotic and inflammatory pathways. Mitochondrial quality control (MQC) has been demonstrated to play an important role in the maintenance of mitochondrial homeostasis. Different factors can induce MQC, including mitochondrial DNA damage, proteostasis dysfunction, and mitochondrial protein translational inhibition. MQC constitutes a complex signaling response that affects mitochondrial biogenesis, mitophagy, fusion/fission and the mitochondrial unfolded protein response (UPRmt) that, together, can produce new mitochondria, degrade the components of the oxidative complex or clearance the entire organelle. In pulmonary fibrosis, defects in mitophagy and mitochondrial biogenesis have been implicated in both cellular apoptosis and senescence during tissue repair. MQC has also been found to have a role in the regulation of other protein activity, inflammatory mediators, latent growth factors, and anti-fibrotic growth factors. In this review, we delineated the role of MQC in the pathogenesis of age-related pulmonary fibrosis.

## 1. Introduction

Idiopathic pulmonary fibrosis (IPF) represents one of the most aggressive and irreversible lung diseases, usually diagnosed in the fifth decade of life, carries a very poor prognosis, an unknown etiology and limited therapeutic options [1]. Metabolic alterations seen in aging have also been found in the lungs of patients with IPF, raising the possibility that aging may contribute to the IPF pathogenesis [2]. Alveolar epithelial cells (AEC) of IPF lungs have shown to exhibit short telomeres, predisposing these cells to apoptosis, leading to abnormal parenchymal architecture, dysfunctional re-epithelialization and exaggerated inflammatory reaction that contributes to the development of fibrotic scarring. In addition, fibroblast of IPF lungs showed abnormal cellular morphology with reduced mitochondrial mass, disrupted membranes and severe ruptured cristae, prompting the fibroblasts to maladaptive responses to stress after injury and increasing susceptibility to fibrosis. Among these alterations, mitochondria dysfunction is recognized as a major hallmark that accounts for the predilection to fibrosis in aging [3]. While mitochondria were initially viewed as just the powerhouse of the cell, advances in the field has allowed us to understand the multiple roles of this organelle. Mitochondria are involved in the heme biosynthesis, intracellular calcium regulation, ATP-production and fatty acid synthesis. Thus, to guarantee adequate organelle function and maintenance of intracellular homeostasis, mitochondria rely on quality control pathways: mitochondrial biogenesis, fusion/fission, mitophagy and the mitochondrial unfolded protein response (UPRmt). Biogenesis of mitochondria allows proper cellular homeostasis, it relies on the action of PGC-1a, the major regulator of this process and its activation via different proteins (AMPK, SIRT1, MAPK and CREB). Fusion/Fission represents the cornerstone of mitochondria dynamics by controlling the cellular bioenergetics and mitochondrial networks via the actions of Drp1, Fis1, Mff, Mfn1 and Mfn2. Selective removal of damaged and dysfunctional mitochondria requires the coordinated action between PINK1/Parkin pathway activation. Accumulation of unfolded proteins inside the mitochondria leads to activation of the mitochondria unfolded protein response (UPRmt) with the goal of promoting repair, recovery and restore mitochondrial proteostasis. Activation of these machineries allows maintenance and regulation of this organelle metabolism, biogenesis, ROS production and mitochondrial DNA (mtDNA) damage repair [4,5]. Disruptions of these processes can subsequently lead to the accumulation of dysfunctional mitochondria, alter the intracellular environment and contribute to the development of age-related lung fibrosis [6]. While the exact triggering injury leading to irreversible fibrosis seen in IPF is unknown, dysregulation of the mitochondrial quality systems suggests a clear pathogenic role that could explain the metabolic dysregulation, proteostatics alterations and decline in mitochondrial function of patients with IPF. This review explores the mitochondrial quality control pathways, its association with the development of age-related lung fibrosis and describes new potential therapeutic targets.

## 2. Mitochondrial Quality Control Pathways

### 2.1. Mitochondrial Biogenesis

**Figure 1:**
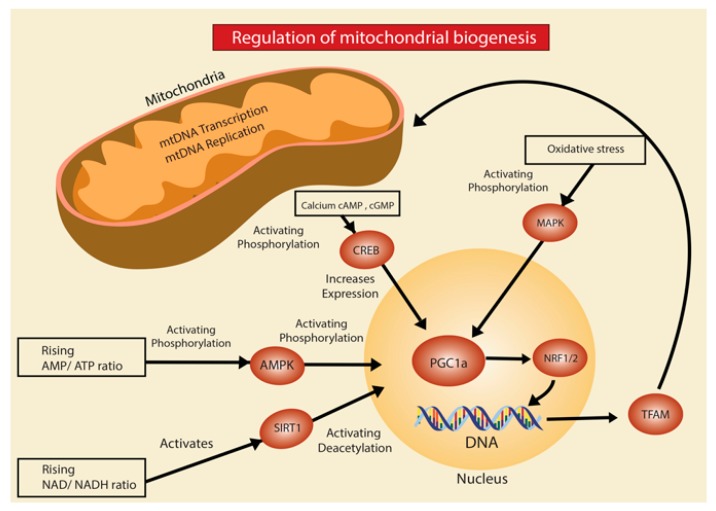
Regulation of mitochondria biogenesis. Activation of different signaling pathways, such as AMPK, SIRT1, CREB, MAPK has been associated with mitochondria biogenesis by increasing PGC-1a gene transcription. PGC-1a represents the major co-transcriptional factor that regulates mitochondria biogenesis by activating the nuclear respiratory factor 1 (NRF1) and nuclear respiratory factor 2 (NRF2) which leads to increase expression of mitochondrial transcription factor (Tfam), driving transcription and replication of mitochondria DNA (mtDNA).

Mitochondria biogenesis is a complex and tightly regulated process. Mitochondrial biogenesis is defined as increased growth in the number of mitochondria from the growth and division of pre-existing mitochondria [7]. The major regulator of this process, co-transcriptional factor PGC-1a, has the ability to activate different transcription factors such as nuclear respiratory factor 1 (NRF-1) and nuclear respiratory factor 2 (NRF-2) [8]. These transcription factors, in turn, increase the expression of mitochondrial transcription factor (Tfam), which drives transcription and replication of mitochondrial DNA (mtDNA) (Figure 1). In response to injury or proteostatic stress, mitochondria biogenesis is stimulated to increase cellular energy production through AMP-activated protein kinase (AMPK), a protein considered another major regulator of mitochondrial biogenesis when energy levels inside the cell decrease [9]. Furthermore, the reduction of AMPK and PGC-1a are a major contributing factor for the mitochondrial dysfunction evidenced in the bleomycin-induced lung fibrosis model [10,11]. Another mechanism implicated, is through upregulation of the ROS-producing enzyme NADPH oxidase-4 (Nox4) which represses NRF-2 and Tfam leading to diminished biogenesis [12]. In addition, experimental models have suggested a direct association between DNA damage causing activation of injury sensors poly(ADP-ribose) polymerase 1 (PARP-1) and p53 which diminish mitochondrial biogenesis by inhibiting the expression of PGC-1a [13,14].

Mitochondria biogenesis is closely associated with the mTOR signaling, activation of this pathway directly leads to increased expression of PGC-1a. Studies have demonstrated that activation of the mTORC complex 1 (mTORC1) upregulates biogenesis by preventing the binding of the eukaryotic translation initiation factor 4E (eIF4)-binding proteins (4E-BP) to their targets. As a result, translation of nuclear-encoded mitochondrial proteins of complex V, complex I and Tfam takes place [15]. Furthermore, mTOR signaling is a highly regulated pathway, proper function and quality control relies on the function of the proteasome and ubiquitin-ligases activity to accomplish protein synthesis required for mitochondria biogenesis. The actions of mTORC1 are carried out within minutes of activation, coordinating the inhibition of autophagy, promoting protein synthesis, suppressing protein turnover and activating the transcription factor SREBP1 [16,17]. The underlying mechanisms of how proteolysis is initially inhibited are unknown It is possible that phosphorylation of E3-ligases by mTORC1 inhibits ubiquitination and subsequently proteolysis by the 20S subunit of the proteasome. During early response, inhibition of protein degradation allows the proper stability of newly generated ribosomes and translation initiation. In the following hours, SREBP1 leads to transcriptional activation of the nuclear factor erythroid-derived 2-related factor (Nrf1, also known as NFE2L1) which promotes proteasome gene expression as a delayed response [18]. Therefore, increased protein degradation is seen in the late stages of the mTORC1 response. Conceivably, the delayed production of proteasomes creates an efficient clearance of proteins to protect the cell from the accumulation of misfolded-cytotoxic proteins and simultaneously maintains an adequate pool of recycled amino acids to continue mitochondria biogenesis. Interaction between mitochondria dysfunction, mTORC1 and the ubiquitin-proteasome system might represent the key element to understand in depth the mechanism that lead to mitochondrial biogenesis dysregulation in age-related lung fibrosis.

Currently, the role of mitochondria biogenesis in age-related lung fibrosis is not fully elucidated. Studies have reported that with aging, mitochondria biogenesis declines due to a reduction in PGC-1a, PGC-1b, AMPK and p-53 through senescence-associated mechanism. (16) Low expression of PGC-1a was evidenced in patients with IPF and fibrotic mouse lungs after bleomycin treatment [19]. In addition, Yu et al. demonstrated that aerosolized thyroid hormone (TH) blunted bleomycin and TGF-B-induced fibrosis in mice. Furthermore, TH was able to restore mitochondrial function and morphology in AECII through PINK1 and PGC-1a actions. [20] However, as we demonstrated in our study, mitochondria biogenesis is increased in the AECII of old mice through the upregulation of the mTORC1/PGC-1 signaling axis. In addition, we found the upregulation of other downstream molecules associated with mitochondria biogenesis, including Tfam and NRF1. Previous reports have demonstrated that an increase in mitochondria biogenesis could lead to cellular senescence by enhancing ROS-mediated damage, as a result of boosting mitochondrial respiration. This is based on the fact that administration of MitoTempo, following bleomycin exposure in AECII, inhibits the mTORC1 response and suppresses the expression of senescence markers. Moreover, chronic activation of the mTORC1/PGC-1 axis could explain the accumulation of large, dysmorphic mitochondria found in the aged mice by over-activating biogenesis and inhibiting mitophagy to remove defective mitochondria [21]. Although many pieces remain unsolved, certainly mTORC1/PGC-1 axis represents a potential therapeutic target in the treatment of age-related lung fibrosis. Further translational studies are needed, nonetheless, current evidence points that quality control of mitochondria biogenesis plays a crucial role in the pathogenesis of age-related lung fibrosis, distortions in its regulation can shift the cell towards senescence and promote inflammation and fibrosis.

### 2.2. Mitochondrial Dynamics

Mitochondria are dynamic organelles in that they require the balance of fission and fusion for proper function and adaptation to cell growth, division, and injury response. Fission and fusion represent the first line of quality control in the setting of proteostatic stress [22,23]. Quality control of fission and fusion is tied to appropriate cellular bioenergetics and homeostasis of mitochondrial networks. Fission is carried by the action of dynamin-related protein 1 (Drp1), fission 1 (Fis1), and mitochondria fission factor (Mff). Fission occurs when Drp1, located in the cytosol or at the endoplasmic reticulum, is recruited to the OMM where it constricts the mitochondria resulting in two separate organelles. In order to achieve this, Drp1 requires the formation of a complex through an interaction with the OMM receptors Fis1 and Mff. Hydrolysis of the Drp1-bound GTP constricts the Drp1 and allows severing of the enclosed membranes resulting in fission. Moreover, mitochondria fusion is carried out by proteins mitofusin1 (Mfn1), mitofusin 2 (Mfn2), and optic atrophy 1 (OPA1). To start this process, proteins Mfn1 and Mfn2, which contain two transmembrane domains in the OMM with a GTPase domain and are oriented towards the cytoplasm, while OPA1 is found in the IMM and has a dynamin-related GTPase capacity. Then, mitochondria lipid bilayer mixing is performed in a Mfn1/Mfn2-dependent manner with a GTP hydrolysis to allow energy for the OMM fusion. Similarly, IMM fusion requires a similar action by OPA1 to allow merging [24,25,26].

**Figure 2:**
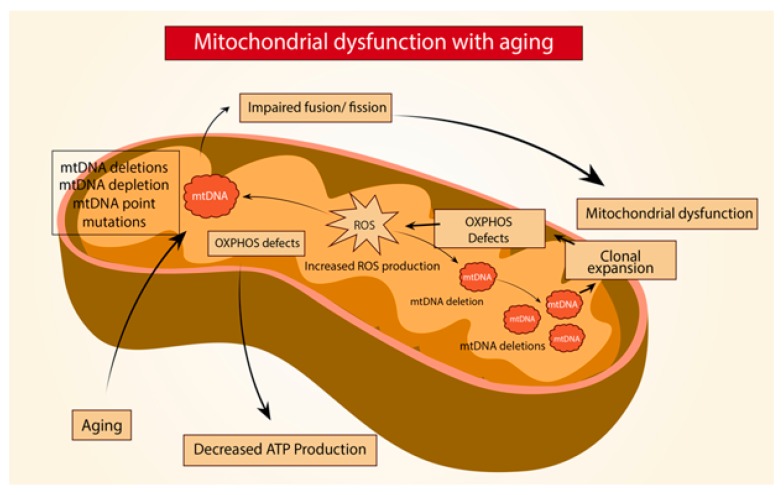
Mitochondria dysfunction with aging. In aging, alterations of the mitochondria DNA (mtDNA) such as deletions, depletion or point mutations can lead to defects in the mitochondria oxidative phosphorylation system (OXPHOS) decreasing ATP production and increasing reactive oxygen species (ROS) production, generating further mtDNA damage, causing impaired fusion/fission and ultimately leading to mitochondrial dysfunction.

Age-related lung fibrosis comprises a very complex physiopathology that involves a constant cycle of injury and repair along with persistent release of pro-inflammatory and pro-fibrotic peptides, which ultimately lead to mitochondrial dysfunction (Figure 2). Mouse models of IPF have shown increased mitochondria fusion. This increase in mitochondria fusion leads to alterations of mitochondrial dynamics by impairing mitophagy and result in the accumulation of dysmorphic mitochondria. To support this, Bueno et al. reported that AECII in old lungs exhibit enhanced expression of OPA1 and Mfn1 with inactivation of Drp1 in addition to impaired mitophagy and a decline in mitochondrial function [19]. Also, one suggested mechanism of Drp1 regulation is carried by the E3 ligase MARCH5 and SUMO-1, where ubiquitination and sumolyation stabilizes or recruits Drp1 to modulate fission [27]. Further, the complex formed by Mdm30 with the ubiquitin ligase Skp1-Cullin-F-box (SCF-Mdm30) regulates the degradation of Mfn1/Mfn2. Similarly, Cilenti et al. determined that the MULAN/MAPL ligase can modulate mitochondrial morphology by promoting Mfn2 degradation [28,29]. Interestingly, Radke et al. demonstrated that proteasome inhibition was capable of augmenting mitochondrial fission [30]. This observation points out a link between the UPS and mitochondrial dynamics. In the aged lung, it is possible that the activity of the proteasome machinery modulates mitochondrial morphology and function by restructuring proteostasis along with regulating fusion/fission. Ultimately, recognition and deeper understanding of the mechanisms involved could lead to new therapeutic approaches targeting mitochondria quality control.

### 2.3. Mitophagy

Mitophagy is a selective autophagy where damaged and dysfunctional mitochondria are degraded. This maintains a homeostatic environment between synthesis and degradation of cellular organelles and proteins, where byproducts are sent to be recycled in other metabolic pathways [31]. Mitophagy is crucial in the quality control of mitochondria not only at the organelle level but also at a cellular one, given its association with senescence, apoptosis, and necroptosis. Impairments in mitophagy have been associated with multiple diseases, including age-related pulmonary fibrosis [6,32].

In the healthy mitochondria, mitophagy relies in the action of PTEN-induced putative kinase 1 (PINK1), a serine/threonine kinase which is continuously imported to the inner membrane (IM) to be cleaved and degraded by the mitochondria-specific proteases presenilin-associated rhomboid-like protein (PARL) and mitochondrial processing peptidases (MPP) [33,34,35]. In the setting of mitochondrial damage, PINK1 acts as the initial sensor to activate mitophagy, where stress-induced membrane depolarization inactivates PARL and MPP. This leads to the stabilization and accumulation of PINK1 on the outer-mitochondrial membrane (OMM), which allows the kinase domain of PINK1 to phosphorylate OMM proteins, in addition to recruitment and activation of the E3-ubiquitin ligase Parkin. Once Parkin is activated, it can polyubiquitinate proteins to the OMM, including voltage-dependent-anion channel 1 (VDAC1), mitofusin 1 (Mfn1), mitofusin 2 (Mfn2) and mitochondria1 rho 1 (MIRO) in order to be sub-sequentially phosphorylated by PINK1. The ubiquitination of these proteins on the OMM leads to the formation of the autophagosome by promoting a bridge with microtubule-associated protein 1 light chain 3 (MAP1LC3/LC3) on phagophores through adaptor protein SQSTM1/p62. Additionally, the ubiquitination of Mfn1 and Mfn2 results in fission, fragmentation, and subsequent degradation with mitophagy through the formation of the autophagosome [36,37,38].

Mitophagy can also occur independently of Parkin [39,40,41,42,43]. Other receptors such as Bcl2/adenovirus protein-interacting protein 3 (BNIP3), NIX, Cardiolipin, and Fun14 domain containing 1 (FUNDC1) contain an LC3 interacting region which promotes mitophagy by the formation of an autophagosome. Moreover, further mitochondrial E3 ubiquitin ligases have been identified to play a role in Parkin-independent mitophagy are SMAD-specific E3 ubiquitin ligase 1 (Smurf1) and Mul1 [44,45]. Interestingly, Smurf1 relies on its C2 domain for the autophagosome formation instead of the ubiquitin-ligase activity to interact with SQSMT1/p62, while Mul1 overexpression increases mitochondrial ubiquitination and fission leading towards mitophagy [46,47].

Evidence suggests that impairments in the PINK1/Parkin pathway play a key role in the pathogenesis of age-related pulmonary fibrosis [19,48]. A decrease in PINK1, a decrease in autophagy markers, and an increase in mitochondria mass have been associated with the aging and lungs of IPF patients. In AECII from IPF lungs, mitochondria have shown dysfunction, evidenced by an enlarged and dysmorphic structure with severely ruptured cristae [49]. This suggests that accumulation of dysfunctional mitochondria, in this case, is a consequence of impaired mitophagy, especially in highly fibrotic areas. Thus, PINK1 is a crucial mitochondrial quality control regulator, not only for mitophagy but also as a key element in mitochondrial dynamics. PINK1-knockdown increases the expression of pro-fibrotic peptides, such as TGF-B1 and TGF-B2 in AECII after tunicamycin and bafilomycin administration [50,51]. In addition, PINK1-deficienct mice exposed to intratracheal instillation of bleomycin had impaired activity of mitochondrial ETC complex I and IV activity leading to mitochondrial dysfunction, along with an increased susceptibility to develop lung fibrosis through upregulation of cytokine TNFα and downregulation of cytokine IL-10 [19,52]. Moreover, low PINK1 expression correlates with high expression of apoptotic markers, including caspases. This finding may be attributed to a combination of multiple elements. Evidence shows that dysmorphic/dysfunctional mitochondria have reduced transmembrane potential, decreased ETC activity, increased ROS production and increased mitochondrial permeability transition pore, all of which could lead to activation of pro-fibrotic events in the AECII.

Interestingly, mitophagy homeostasis can be impaired by the upstream effects of increased ER stress [53,54]. Lung fibrosis has been associated with high ER stress markers. This inappropriate response to proteostasis alterations leads to the downregulation of PINK1 in AECII through the expression of ATF3, which acts as a transcriptional repressor of PINK1 [51,55,56]. It has been reported that patients with IPF have decreased levels of Parkin expression in isolated lung fibroblasts and myofibroblasts. In addition, Parkin-deficient mice showed an exacerbation of lung fibrosis in the bleomycin-induced model. Kobayashi et al. reported that impaired mitophagy by Parkin deficiency induces activation of platelet-derived growth factor receptor (PDGFR)/mammalian target of rapamycin (mTOR) signaling, prompting differentiation and proliferation of myofibroblast [57]. Furthermore, Yu et al. linked a potential therapeutic and anti-fibrotic effect of thyroid hormone (TH). In this study, aerosolized TH resolved fibrosis in two mice models by promoting biogenesis, improving mitochondrial bioenergetics, and decreasing the apoptosis of AEC in a PPRGC1A or PINK1 dependent manner. This finding represents a novel approach to reversing fibrotic changes, nonetheless the exact molecular mechanism is still yet unclear, especially when previous studies have reported reduced expression of both PINK1 and PPGC1A in IPF lungs [20]. The integrity of the proteins involved in mitophagy is tightly regulated, for instance, Parkin is regulated by the UPS through its ubiquitin-like domain that has a high affinity for the subunit Rpn13 of the 26S proteasome. This offers proteasomal degradation of outer-mitochondrial membranes (OMM) and Parkin degradation once mitophagy has ended, ensuring proper quality control of this process [58]. In contrast, human IPF lungs have impaired proteasome activity which leads to the accumulation of misfolded and aggregated proteins in the ER, suggesting that the decline in mitophagy evidenced in IPF lungs could be associated with high levels of ER stress and altered activity of the proteasome [59]. Together, these observations highlight that quality control of this pathway is diminished in lung fibrosis. Mitophagy represents an adaptive response and a protective role against the development of fibrosis, suggesting manipulation of this pathway as a potential pharmacological target.

### 2.4. Mitochondrial Unfolded Protein Response (UPRmt)

**Figure 3:**
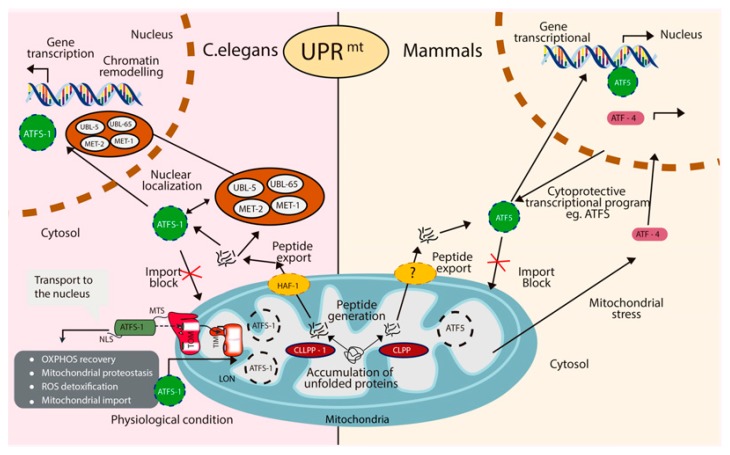
Mitochondria unfolded protein response (UPRmt). In both, mammals and C. elegans, activation of the UPRmt is caused by accumulation of unfolded proteins inside of the mitochondria matrix. Proteolysis of impaired proteins is carried by the effects of the ClpP protease. In C. elegans, export of these peptides to the cytosol is accomplished by HAF-1 which activates ATFS-1 (in C. elegans) or ATF5 (in mammals) to allow nuclear translocation and initiate transcription of this pathway genes. Physiologically, to prevent unnecessary activation of this signaling, ATFS-1 is imported into the mitochondria via the mitochondrial-targeting-sequence (MTS), formed by the translocase of the outer membrane (TOM) and the translocase of the inner membrane (TIM) to allow degradation of ATFS-1 by the protease LON. Furthermore, in C. elegans, translocation to the nucleus of the proteins UBL-5, LIN-65, MET-1 and MET-2 upon UPRmt activation facilitates the binding of ATFS-1 by chromatin remodeling. Ultimately, activation of ATFS-1 and ATF5 induces genes to promote OXPHOS recovery, ROS detoxification, expression of mitochondrial protein import components and upregulation of chaperones and proteases to re-establish mitochondria proteostasis.

The UPRmt is a stress response pathway that promotes repair and recovery in several conditions, such as mitochondria DNA defects, ROS detoxification, and perturbations in mitochondrial proteostasis, protein import machinery or mitochondrial translation. Currently, the molecular mechanism of the UPRmt has been more broadly described in the model organism *Caenorhabditis elegans* (*C. elegans*) than in mammals. In *C. elegans* the master regulator of the UPRmt is ATFS-1 [5,60]. However, it is unclear who the master regulator of the UPRmt is in mammalians. Three possible homologs of ATFS-1 have been identified; ATF4, ATF5, and CHOP. In *C. elegans*, under favorable conditions, ATFS-1 is localized to the mitochondria, where it makes its way to the mitochondrial matrix and is degraded by the protease, LONP1 [61,62,63]. However, in the presence of mitochondrial stress, such as unfolded proteins in the matrix, ATFS-1 will translocate to the nucleus to transcribe UPRmt genes. Unfolded proteins in the matrix are cleaved by CLPP-1. The cleaved peptides are shuttled out of the mitochondria by the transporter HAF-1, located in the intermembrane space (IMS). Accumulation of cleaved peptides prevents mitochondrial transport by inhibiting the action of the translocase of the outer membrane (TOM) and the translocase of the inner membrane (TIM). As a result, the leucine zipper transcription factor ATFS-1 is translocated to the nucleus and initiates transcription of genes such as chaperones, proteases, OXPHOS complexes, and protein import components to promote recovery [64]. In mammals, it is believed that the UPRmt response is regulated by the actions of three bZIP transcription factors, CHOP, ATF4 and ATF5. Interestingly, studies have suggested that each of the transcription factors might play different roles during the response and that activation obeys different stimuli. For instance, ATF5 is required to increase the expression of mitochondrial chaperones and proteases while ATF4 levels were upregulated in response to mtDNA depletion [65]. At the present time, the regulatory mechanism of ATF5, CHOP or ATF4 are not completely understood, different from what it is known in *C. elegans* with the actions of LONP1 and CLPP-1. In addition, it is not clear how the three transcription factors coordinate the signaling during the pathway or if activation of each one can occur independently. Further investigations are needed to understand the different regulatory components of the UPRmt in order to fully elucidate its role in diseases associated with mitochondrial dysfunction (Figure 3).

The above mentioned is considered the canonical UPRmt pathway, but there are other types of UPRmt. These other pathways have been described as the UPRmt sirtuin axis, UPRIMS/ERα axis, and the UPRmt translational axis [66]. While the canonical UPRmt axis aims at restoring proteostasis through increasing the mitochondria’s ability to fold proteins correctly, the other axis uses other methods such as stopping translation or removing aberrant proteins. Increased oxidative stress causes damage to mitochondrial lipids, proteins and mtDNA, resulting in proteotoxic stress in the mitochondria. A recent study by Papa et al. described the role of the mitochondrial deacetylase Sirt3 in the UPRmt. Proteotoxic stress activates Sirt3 to coordinate the antioxidant machinery of the mitochondria by activating the transcription factor FOXOA3 which subsequently increases the expression of the mitochondria superoxide dismutase MnSOD and catalase. Moreover, SirT3 actions are required to maintain mitochondrial integrity during proteotoxic stress independently of CHOP expression [67,68]. Also, inhibition of Sirt3 leads to the downregulation of mitophagy markers and the activation of apoptotic markers, suggesting a major role in the clearance of damaged or dysfunctional mitochondria [69]. Furthermore, patients with IPF have increased acetylation of Sirt3 in AEC, leading to decreased activity of this enzyme and increase acetylation of MnSOD and OOG1 in the lung tissue. Jablonski et al. showed that bleomycin exposure to the Sirt3-/- mice augmented oxidant-induced mtDNA damage, apoptosis, and lung fibrosis while mice overexpressing Sirt3 had preserved mtDNA and were protected from bleomycin-induced lung fibrosis, highlighting Sirt3 as key element in maintaining the integrity of the AEC-mtDNA and a potential therapeutic target by preventing the mtROS injury and apoptosis in IPF [70]. In sum, these findings show the critical role of Sirt3 as a regulator of mitochondrial network integrity to promote recovery and prevent dysfunction separately from the actions of the main transcription factors that drive the UPRmt. This pathway, distinct from the canonical pathway, is referred to as the UPRmt-sirtuin axis.

**Figure 4:**
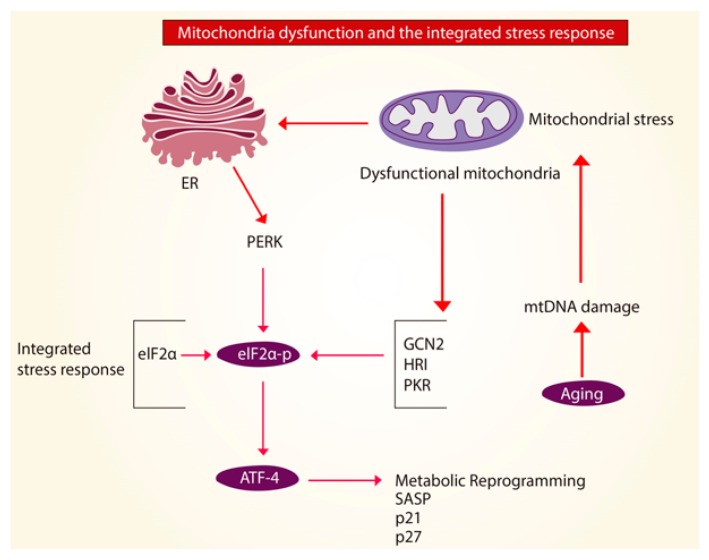
Mitochondria dysfunction and the integrated stress response (ISR). In aging, it is possible that mitochondrial stress and mitochondria dysfunction caused by mitochondria DNA (mtDNA) damage activates the integrated stress response (ISR) leading to phosphorylation of the eukaryotic translation initiation factor 2 subunit 1 (eIF2α) by the kinases GCN2, PERK, HRI and PKR. This causes activation of the transcription factor 4 (ATF4) to allow protein recovery and promote cellular homeostasis. Overexpression of ATF4, can modulate the actions of p21, p27, drive the senescence-associated secretory phenotype (SASP) and stimulate a metabolic reprogramming in the cell that could predispose to fibrotic disease.

A halt or interference of translation is a hallmark of the UPRmt translational axis, but also of the integrated stress response (ISR). The ISR is activated under cell stress that is not solely dependent on the mitochondria. The ISR could be activated as a response to excessive ROS or amino-acid depletion. Some studies suggest that in order to have UPRmt activation, the ISR must also be activated, however, the exact mechanism is still unknown. ISR responses are carried by actions of the kinases which phosphorylate the eukaryotic translation initiation factor 2 subunit 1 (eIF2α). The four kinases known to phosphorylate eIF2α are GCN2, PERK, HRI, and PKR. Phosphorylation of eIF2α interferes with the formation of the translation initiation complex. Thus, phosphorylation of eIF2α inhibits protein translation in the cytoplasm to reduce folding load on mitochondrial chaperones and simultaneously allowing translation of ISR-specific mRNAs, such as ATF4 [71,72,73,74]. While during acute mitochondrial stress, activation of the ISR enables protein recovery the effects of chronic activation of the ISR-ATF4 pathway are yet to be elucidated. Studies showed that increased expression of ATF4 facilitated the progression of age-related diseases by modulating the actions of p21 and p27 [65,75]. Hence, suggesting that mitochondrial dysfunction in aging could lead to chronic activation of the ISR-ATF4 pathway and overexpression of ATF4 might in fact, be harmful in this setting (Figure 4). Although the exact mechanism by which mitochondrial dysfunction promotes chronic ISR-ATF4 activation has not yet been determined, blockage of this pathway opens a new gate for pharmacological inhibitors to treat age-related diseases.

### 2.5. Mitochondrial Quality Control as Therapeutic Target in IPF

The growing evidence of the mitochondria role in the development of IPF has established new opportunities to create therapeutic strategies that target the turnover and dynamics of this organelle. For instance, studies suggest that hormonal modulation of mitochondrial function by 17b-estradiol (E2) through nuclear or mitochondrial ER (Estrogen receptor) can induce antioxidant responses and activation of NRF1/2, Tfam and PGC-1a for mitochondrial biogenesis [76,77,78]. Alternatively, ER-a receptor expression was upregulated in IPF lungs and pharmacologic blockage of this receptor lead to attenuation of fibrosis after bleomycin exposure, presumably by downregulation of the pro-fibrotic pathway carried by Smad2 [79,80]. Further, patients with IPF were reported to have disproportionally decreased synthesis of dehydroepiandrosterone (DHEA), a prohormone linked to antifibrotic properties by decreasing fibroblast proliferation, TGF-B1 collagen production and promoting fibroblast apoptosis. The effects of DHEA in fibroblast cell death resulted from the release of mitochondrial cytochrome C into the cytosol and ultimately activation of caspase 9 [81,82,83]. Moreover, the use of active thyroid hormone T3 has been suggested as potential therapy in lung fibrosis, Yu et al. reported that in mice models of pulmonary fibrosis administration of aerosolized T3 offered regenerative properties by inducing PGC-1a and PINK1, leading to mitochondrial biogenesis and restoration of mitochondrial function and attenuation of apoptosis [20].

Additionally, mitochondria-targeted antioxidant agents such as MitoQ might represent a potential therapy. MitoQ, acting as an ROS-scavenger within the mitochondria decreased the expression of TGF-B1 and NOX4 in fibroblast of IPF patients, attenuating inflammation and collagen deposition [84,85]. It is well known that PINK1 deficiency in AEC of IPF lungs represents a major factor triggering the mitochondrial dysfunction seen in these cells. Thus, agents that potentiate the activity of PINK1 can represent a therapeutic option. The neo-substrate kinetin triphosphate (KTP) showed to increase the activity of PINK1, Parkin and lower apoptosis markers, establishing this compound as a potential drug for these patients [86]. Similarly, the induction of the mitochondrial protein SIRT3 represents an attractive therapeutic option giving its protective effects against injury and fibrosis. A pharmaceutical compound known as Hexafluoro showed to induce the expression of SIRT3 in mice treated with bleomycin, lessening the development of lung fibrosis by reducing the expression of collagen 1, a-SMA and fibronectin through TGF-B1 inhibition [87]. Furthermore, mitochondrial quality control also relies on the presence of enzyme ubiquitylation to clear damaged mitochondrial proteins. This process is negatively regulated by de-ubiquitinating enzymes (DUBs) which inhibit mitophagy by removing the ubiquitin tags added by Parkin. Evidence suggests that the deletion of the de-ubiquitinate USP30, found in the mitochondrial outer membrane, might represent a novel therapeutic target that could enhance mitophagy and augment substrate ubiquitination [88,89]. Taken together, pharmacological modulation of mitochondrial quality control that aims biogenesis, mitophagy and fission/fusion could lead to the prevention of mitochondrial dysfunction and ultimately, fibrosis. Multiple mechanisms of actions have been proposed for the different drugs, nonetheless, collective efforts are still required to fully elucidate the specific molecular mechanism by which many of the suggested compounds exerts their effect with the goal of providing high-quality therapies that can impact survival in patients with this condition.

## 3. Mitochondria in Age-Related Lung Fibrosis

In aging, different models have shown similar decreases in mitochondrial function [90]. Multiple studies have pointed out that chronic activation of the UPRmt correlates with increased longevity in *C. elegans* and mice. However, this activation brings a variety of changes to the mitochondria network including increased fragmentation, and decreased oxygen consumption and ATP production. These changes could represent a protective metabolic response to allow mitochondria repair and as a result prolonged cell survival, or conversely, serve as the initiating factor for the development of aged-related diseases. Different regulators of the UPRmt have been found to be altered with aging. For instance, LONP1 expression was reported to be reduced in lung senescence-fibroblast which lead to the accumulation of oxidized proteins following hydrogen peroxide administration [91,92,93]. Furthermore, senescence-fibroblasts were shown to have altered mitochondrial mass, suggesting that perhaps this morphologic change is partially a result of the reduction in the activity of LONP1 with aging, and could also serve as another explanation for the abnormal morphology observed in the mitochondria of IPF patients [94,95]. Several reports have demonstrated reduced activity of CLPP-1 in aged cells. As a result of deficient CLPP-1, cells were shown to have numerous alterations, such as a higher amount of ROS, decreased ATP production and downregulation of fusion markers, establishing a link between protease abnormalities, energy metabolism and mitochondrial dynamics [96,97]. In addition, CLPP-1-deficient aged cells were found to have an upregulation of mTORC1 signaling markers, suggesting that the increased activation of the mTORC1/PGC-1 axis in aged-AECII reported in our study could be a response to an upstream alteration in the mitochondrial matrix proteases [98,99].

In mammals, efficient regulation of the transcription factors ATF5, ATF4 and CHOP is crucial to develop proper mitochondria-to-nucleus communication during UPRmt activation, and only a few studies have described the association of these transcription factors with age-related diseases. Firstly, ATF5 has been described as an activator of the mTORC pathway resulting in autophagy inhibition along with inhibition of apoptosis through the expression of BCL-2 anti-apoptotic factor [100,101]. In addition, loss of ATF5 leads to significant apoptosis following thapsigargin (Tg) administration to induce-ER stress [102]. Altogether, these findings posit ATF5 as a key pro-survival mediator during proteotoxic damage, a hallmark of age-related diseases. Moreover, multiple aging mice models and AECII from IPF patients have demonstrated increased transcription of ATF4 and CHOP. Studies suggest that ATF4 induces CHOP, which contributes to the induction of GADD34, the main regulator of a common adaptive pathway; termed the integrated stress response (ISR), to restore cellular homeostasis in response to ER stress, depletion of amino-acids and oxidative stress [103,104,105]. Activation of this pathway through increased ER stress has been associated with chronic injury and diseases that ultimately lead to pulmonary fibrosis, such as STFPC-mutations in familial IPF and Hermansky-Pudlak syndrome, suggesting a link between UPRmt and ER stress as a possible trigger mechanism of the aberrant fibrotic pattern in lung fibrosis [106,107]. Likewise, the role of CHOP in the development of lung fibrosis has been described in several reports. In the mouse model of bleomycin-induced pulmonary fibrosis, CHOP expression was found to be upregulated primarily in AECII of highly fibrotic areas, while using the same model, CHOP-deficient mice reported a marked reduction of fibrotic areas and significant lower apoptotic markers [108]. Upregulation of apoptosis-related genes, including GADD45A and BNIP3L, are modulated by CHOP expression in AEC. Interestingly, activation of GADD45A is associated with p53 upregulation as a response of DNA damage and apoptosis induction, increased expression of this protein was found in AEC in IPF [109,110,111,112]. Together, these findings indicate that CHOP plays a key role in the promotion of fibrotic remodeling following chronic injury and that possibly these effects are triggered by AEC apoptosis, making this transcription factor a target for future therapeutic prospects.

## 4. Conclusions

Currently, there are a limited number of marginally effective treatment options for patients with progressive forms of fibrotic lung disease, emphasizing the need for further mechanistic insight and translational progress. Evidence that mitochondrial dysfunction initiates fibrotic response is especially strong in IPF, but the mechanism linking mitochondrial quality control to aberrant repair remains elusive. Future studies determining how dysregulation of mitochondrial quality control contributes to the onset or progression of IPF will ultimately be important for advancing understanding of disease and laying the foundation for new and more effective treatments.
